# The relation between blood pressure components and left atrial volume in the context of left ventricular mass index

**DOI:** 10.1097/MD.0000000000009459

**Published:** 2017-12-29

**Authors:** Marta Rojek, Marek Rajzer, Wiktoria Wojciechowska, Jerzy Gąsowski, Tomasz Pizoń, Danuta Czarnecka

**Affiliations:** a 1st Department of Cardiology, Interventional Electrocardiology and Arterial Hypertension, Jagiellonian University Medical College, Kraków, Poland; b Medical Faculty, Dresden University of Technology, Dresden, Germany; c Department of Internal Medicine and Gerontology, Jagiellonian University Medical College; d Department of Observational and Internal Medicine, University Hospital, Kraków, Poland.

**Keywords:** arterial stiffness, blood pressure steady and pulsatile components, left atrial volume, left ventricle mass

## Abstract

Left atrial enlargement (LAE) is a risk factor for cardiovascular complications and death. In hypertensive patients, LAE is usually due to left ventricular (LV) hypertrophy and diastolic dysfunction. We aimed to identify factors associated with LAE in patients with increased and normal left ventricular mass index (LVMI) with reference to pulsatile and steady components of blood pressure (BP).

The study was carried out as a cross-sectional observation. In a group of inhabitants of suburban area of Cracow, Poland, we measured office, ambulatory and central BP, carotid-femoral pulse wave velocity (PWV), as well as echocardiographic indices and gathered anthropometric data, information on habits and relevant medical history. Further, with division according to sex-stratified dichotomised LVMI, we performed correlation analysis to identify possibly significant relations between measures of left atrial volume and other studied parameters. We also fitted regression models in order to assess the respective value of steady and pulsatile BP components as factors related to measures of left atrial volume.

The mean age of 205 patients (136 females—66%) was 53.6 ± 8.3 years. We found higher values of PWV, office, ambulatory and central BPs in the group of LVMI above median value. This group had also greater left atrial volume index (LAVI), which correlated with LVMI (*r* = 0.36, *P *< .001) and ratio of early diastolic mitral peak flow velocity to early diastolic mitral annulus mean velocity in tissue Doppler imaging (*E*/*e*′) (*r* = 0.24, *P *= .04).

In the group of LVMI below the median, LAVI correlated with pulsatile and steady BP components. LAVI was independently predicted by mean arterial pressure (MAP) obtained from both ambulatory (MAP_24h_, β= 0.15; *P *= .045) and office measurements (MAP_office_, β = 0.35; *P *= .004), but not by pulse pressure.

LV mass and function are the main determinants of LAVI. However, in persons with lower LV mass, LAVI depends on the steady component of blood pressure, but not pulsatile one. Increased LAVI reflects early changes in response to systemic blood pressure elevation.

## 1 Introduction

Left atrial enlargement (LAE) is a risk factor for paroxysmal supraventricular arrhythmias including atrial fibrillation, systemic embolism and death.^[1–4]^ LA enlargement measured by left atrium volume index (LAVI) was reported to be an independent predictor of major adverse cardiovascular events among patients postacute coronary syndrome.^[5]^ LAVI might also be useful in the prediction of first ischemic stroke and subsequent mortality,^[6]^ as well as mortality in heart failure patients.^[7]^

LA volume is a marker of left ventricle (LV) diastolic dysfunction severity and duration.^[4,8]^ Increased LA volume is mainly the result of impaired LV filling. In patients with arterial hypertension, the latter is a consequence of LV hypertrophy and remodeling. It has been suggested that LV hypertrophy is a link between hypertension and left atrium enlargement.^[9]^ Furthermore, LA enlargement caused by hypertension is often detected earlier than LV hypertrophy or dilatation in the course of hypertensive heart disease.^[10,11]^

Epidemiological studies proved the relation between cardiovascular risk, mortality and morbidity, and the level of both systolic blood pressure (SBP) and diastolic blood pressure (DBP).^[12–14]^ However, it has recently been suggested that new BP parameters, including central aortic BP and carotid-femoral pulse wave velocity (PWV), should also be analyzed due to their impact on cardiovascular outcomes. The pulsatile component of BP has been demonstrated in numerous studies to be involved in the pathogenesis of atherosclerosis and to be an independent marker of cardiovascular risk.^[15–18]^

The pulsatile component, estimated by pulse pressure (PP), represents BP variation and is affected by heart rate, left ventricle ejection fraction (LVEF), and predominantly by large artery stiffness measured by carotid-femoral pulse wave velocity.^[15]^ A stiffened aorta is less able to adapt to the volume of blood ejected by the LV, which increases the amplitude of SBP. The early return of the reflected wave in systole, not in diastole, decreases DBP. The combined effect is a widened PP.^[19]^ The gold standard of large artery stiffness assessment recommended by experts is carotid-femoral pulse wave velocity (PWV).^[20]^ Some studies have indicated a relation of PWV with LA size in patients with hypertension.^[21,22]^

The question of which component of BP, steady represented by mean arterial pressure (MAP) or pulsatile, is a stronger predictor of LA enlargement remains unresolved. The results of the Framingham study indicate that duration of arterial hypertension and level of SBP, which reflects the pulsatile component of BP, in the general population are responsible for the rise in LA size.^[23]^ Several other studies demonstrated a positive relation between PP and LA enlargement.^[24–26]^ There is lack of sufficient data from clinical studies about the relation of MAP to LA volume or size. Therefore, we decided to identify factors associated with LA enlargement in patients with LVMI below and over the median, with particular reference to pulsatile and steady components of blood pressure.

## 2 Material and methods

### 2.1 Study population

This observational, cross-sectional study included 205 subjects (136 females). Participants were recruited from a list of 1851 Morawica and Jeziorzany inhabitants (suburban area of Cracow, Poland) between June 2015 and January 2016. Of 462 residents aged between 40 and 65 years, 120 refused to participate, 45 temporarily moved, and 92 were excluded from the study due to acute or chronic diseases. The exclusion criteria included: heart, liver, kidney or respiratory failure; coronary artery disease. The recruited group is representative for the population in given age range. We obtained anthropometric data, information on habits, and relevant medical history from all patients with the use of a standardized questionnaire.

Arterial hypertension was diagnosed according to the 2013 European Society of Hypertension/European Society of Cardiology (ESH/ESC) Guidelines.^[27]^ Diabetes was diagnosed according to World Health Organization recommendations.^[28]^

The Jagiellonian University Ethics Committee approved the study protocol (decision number: 122.6120.122.2015 issued June 25th 2015).

All participants were informed of the purpose and methodology of the study and gave written consent.

### 2.2 Blood pressure measurements

We performed office blood pressure measurements twice on the nondominant arm after 10 minutes of rest using Omron M5-I device (Omron, Kyoto, Japan) and results were averaged. Systolic blood pressure (SBP_office_), diastolic blood pressure (DBP_office_), pulse pressure (PP_office =_ SBP_office_–DBP_office_) and MAP_office_ were determined. MAP_office_ was calculated according to the formula: MAP_office_ = DBP_office_ + 1/3 PP_office._ Around 24 hours ambulatory blood pressure monitoring (ABPM) was performed with the use of SpaceLabs 90207 device (SpaceLabs Healthcare, Snoqualmie, WA) according to ESH expert recommendations.^[29]^ Measurements were performed every 15 minutes during daily activity (6:00–22:00) and every 20 minutes at night (22:00–6:00). The above-described approach allowed determining 24 hours, day and night values of systolic blood pressure (SBP_24h,_SBP_D_, SBP_N_), diastolic blood pressure (DBP_24h,_ DBP_D_, DBP_N_), pulse pressure (PP_24h,_ PP_D_, PP_N_) and mean arterial pressure (MAP_24h,_MAP_D_, MAP_N_).

### 2.3 Pulse wave analyses

We assessed arterial stiffness parameters by measuring carotid-femoral PWV and components of central pressure: central systolic blood pressure (cSBP), and central pulse pressure (cPP) by applanation tonometry method with the use of SphygmoCor device integrated with relevant analytical software (AtCor Medical, Sydney, Australia). The examination was performed according to expert consensus documents- in supine position, predominantly at the right common carotid and right common femoral arteries, after patient's 10 minutes rest.^[20,30]^ We took 2 measurements and the mean value was included in further analyses.

### 2.4 Echocardiographic measurements

We performed all echocardiographic measurements employing 2 independent examiners by using Vivid 7 ultrasound system (General Electric Healthcare, Milwaukee, WI) equipped with a harmonic 1.7 to 3.4 MHz variable frequency phased-array transducer. The study protocol was in agreement with ESC recommendations.^[31]^ LV mass and LVMI were calculated by using Devereux formula.^[32]^

LA volume was measured using the modified Simpson's method.^[31]^ Maximum LA areas, except for the confluence of pulmonary veins and the left atrial appendage, were traced in apical 2- and 4-chamber views at end systole of the LV. LAVI was calculated as LA volume/body surface area. To assess intraobserver and interobserver variabilities in LAVI measurements, 15 patients were randomly selected and measurements were taken by the main observer at 2 separate occasions and another independent observer. We computed the coefficient of variation as the ratio of the mean difference between repeat measurements to the standard deviation of the paired differences multiplied by 100. Intraobserver and interobserver variabilities were around 4% and 6%, respectively.

The transmitral flow velocity was obtained from the apical long-axis view with the pulse Doppler method and the early diastolic mitral peak flow velocity (*E*), late diastolic mitral peak flow velocity (*A*), and their ratio were calculated.

The mitral annular motion velocity was recorded at the LV lateral and septal wall sites in the apical 4-chamber view by pulsed tissue Doppler echocardiography. The means of peak early diastolic motion velocity (*e*′) at both sites and the ratio of *E* to *e*′ (*E*/*e*′) were determined.

Once all clinical examinations were performed, we divided study group into 2 subgroups based on sex-stratified dichotomized LVMI. The 1 group consisted of patients with LVMI over the median value and the other group of patients with LVMI below the median value.

### 2.5 Statistical analysis

Statistical analyses were performed using STATISTICA 12 software (StatSoft, Inc., Tulsa, OK). The results are expressed as numerical values and percentages for categorical variables and mean values ± standard deviation for continuous variables. Comparisons with unrelated groups were made using Student's *t*-test for continuous variables and χ^2^ for qualitative variables. Analysis of the Pearson correlation coefficient was used to determine the relation between considered variables and LAVI. Independent factors’ influence on LAVI were assessed in univariate and multivariate linear regression models. Differences were considered statistically significant at *P *< .05.

## 3 Results

A total of 205 persons (66% females, 53.6 ± 8.3 years) were included in this study and divided into 2 subgroups according to the sex-specific median LVMI value (i.e., 97 g/m^2^ for women and 110 g/m^2^ for men). Clinical, anthropometric, and demographic characteristics of study groups are presented in Table 1.

**Table 1:**
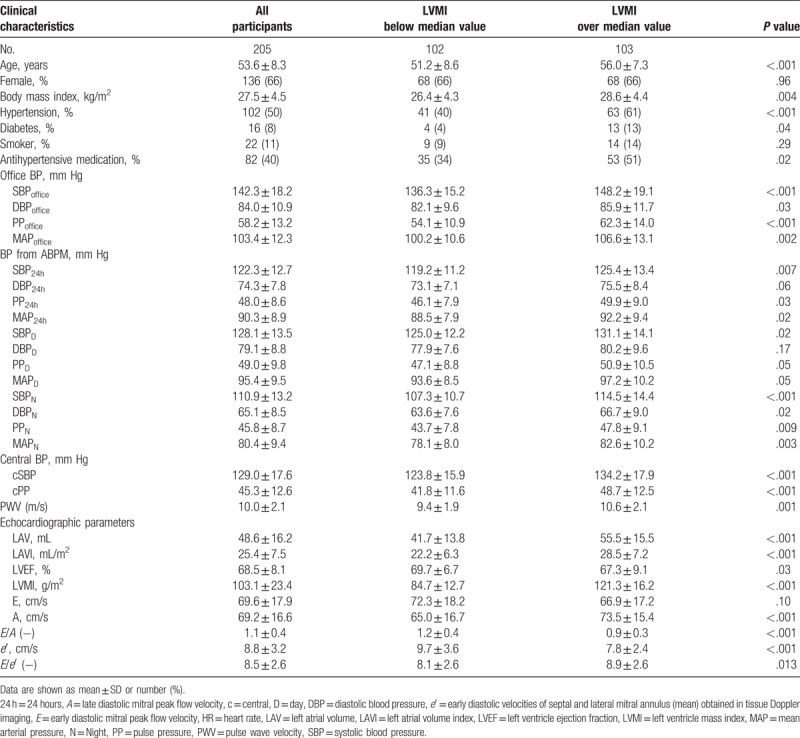
Clinical characteristics of study participants according to sex-specific median of left ventricle mass index (LVMI).

A higher prevalence of arterial hypertension and diabetes, as well as higher body mass index (BMI), was observed in participants with LVMI above median. Those subjects also had higher PWV, central, peripheral, and ambulatory blood pressure, and LAVI (Table 1).

The study population consisted of the subjects with a LVEF within normal range and no signs of regional LV contraction disturbances.

Table 2 summarizes analyses’ results of relation between LAVI and other variables including blood pressure values, arterial stiffness, and echocardiographic parameters in both groups. In the group with LVMI above median value, LAVI correlated positively with LVMI and other echocardiographic parameters including *E*/*e*′, but not with blood pressure parameters and PWV. In the group with LVMI below median value, LAVI correlated positively with blood pressure parameters, PWV, and echocardiographic parameters including LVEF, LVMI, and indices of LV diastolic function.

**Table 2:**
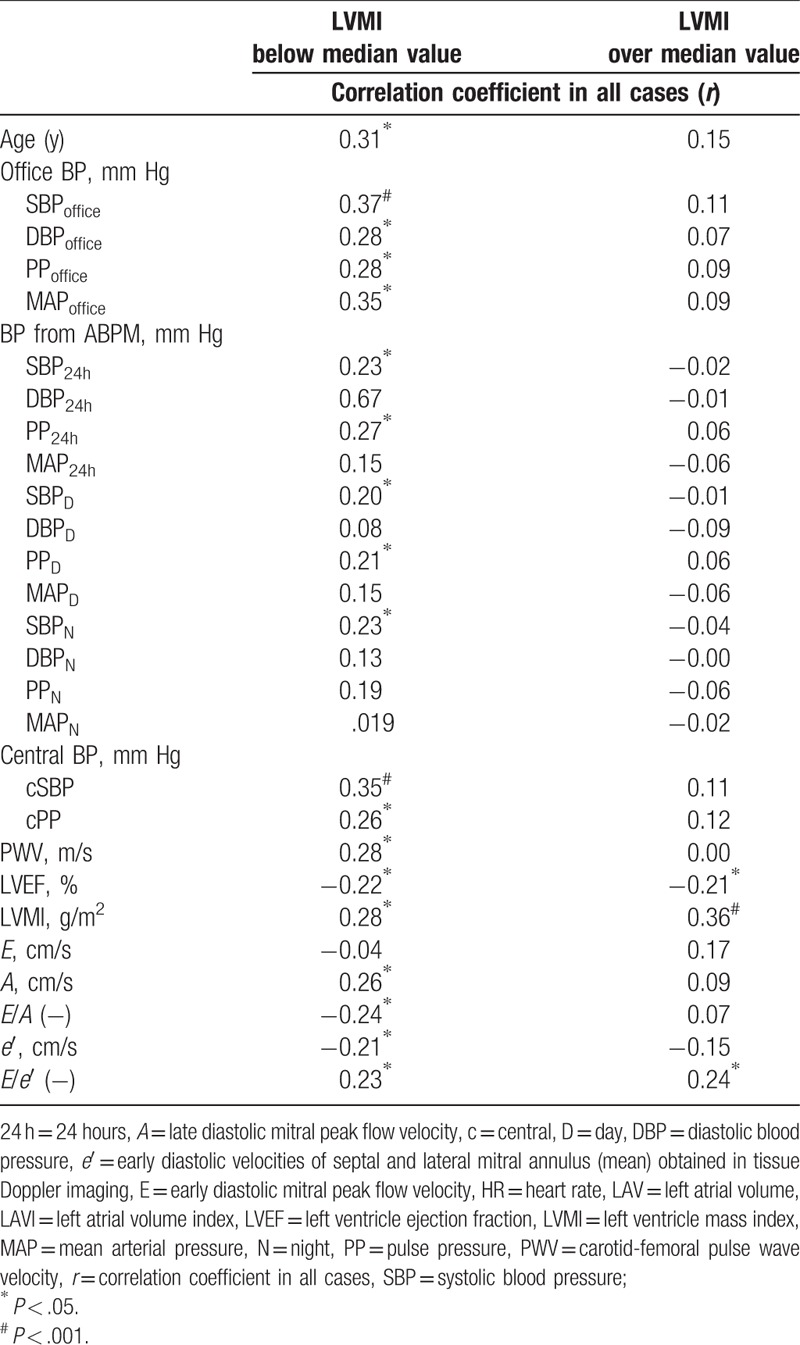
Relation of anthropometric, hemodynamic and echocardiographic measurements with left atrial volume index (LAVI) according to sex-specific median of left ventricle mass index (LVMI) value.

**Figure 1:**
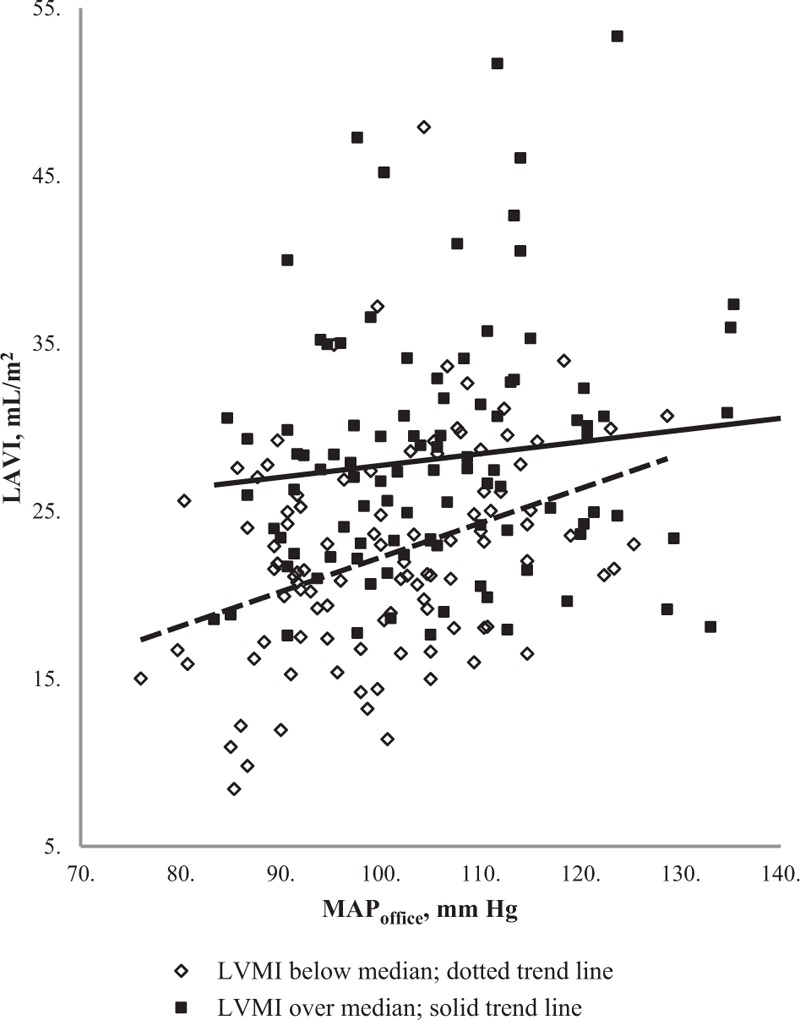
Open rhomb and dotted line represent the relation of left atrium volume index (LAVI) to office mean blood pressure (MAP office) in participants with left ventricle mass index (LVMI) below median value. Model equation: LAVI = 1.73 + 0.21^∗^ MAP_office_; *P* < .001. Full square and solid line represent relation of left atrium volume index (LAVI) to office mean blood pressure (MAP office) in participants with left ventricle mass index (LVMI) over median value. Model equation: LAVI = 20 + 0.07^∗^ MAP_office_; *P* = .22. LAVI- left atrial volume index; LVMI- left ventricle mass index; MAP_office_- office mean arterial pressure. LAV = left atrial volume index; LVMI = left ventricle mass index; MAP_office_ = office mean arterial pressure.

The relation of LAVI with MAP_office_ in study group according to LVMI median value is presented in Figure 1. Significant association is observed only among the participants with lower values of LVMI.

Effect of independent factors (age, gender, PWV, MAP_office_, and MAP_24h_) on LAVI in patients with LVMI below and over the median value was determined by fitting univariate and multivariate linear regression models.

The results of regression analyses are provided in Table 3. Among participants with increased LVMI, we observed no association between LAVI and blood pressure indices. In all evaluated models only age and gender contributed significantly to LAVI value in this group. At the same time, in the group with LVMI below median value, LAVI was influenced by MAP_office_ (β=0.35; *P *= .004) in Model 4 and MAP_24h_ (β=0.15; *P *= .045) in Model 1. Our analyses do not confirm any relation between LAVI and arterial stiffness index-PWV (Model 1 and Model 2) or the pulsatile component of blood pressure-cPP (Model 3 and Model 4) in participants with LVMI below, as well as above the median.

**Table 3:**
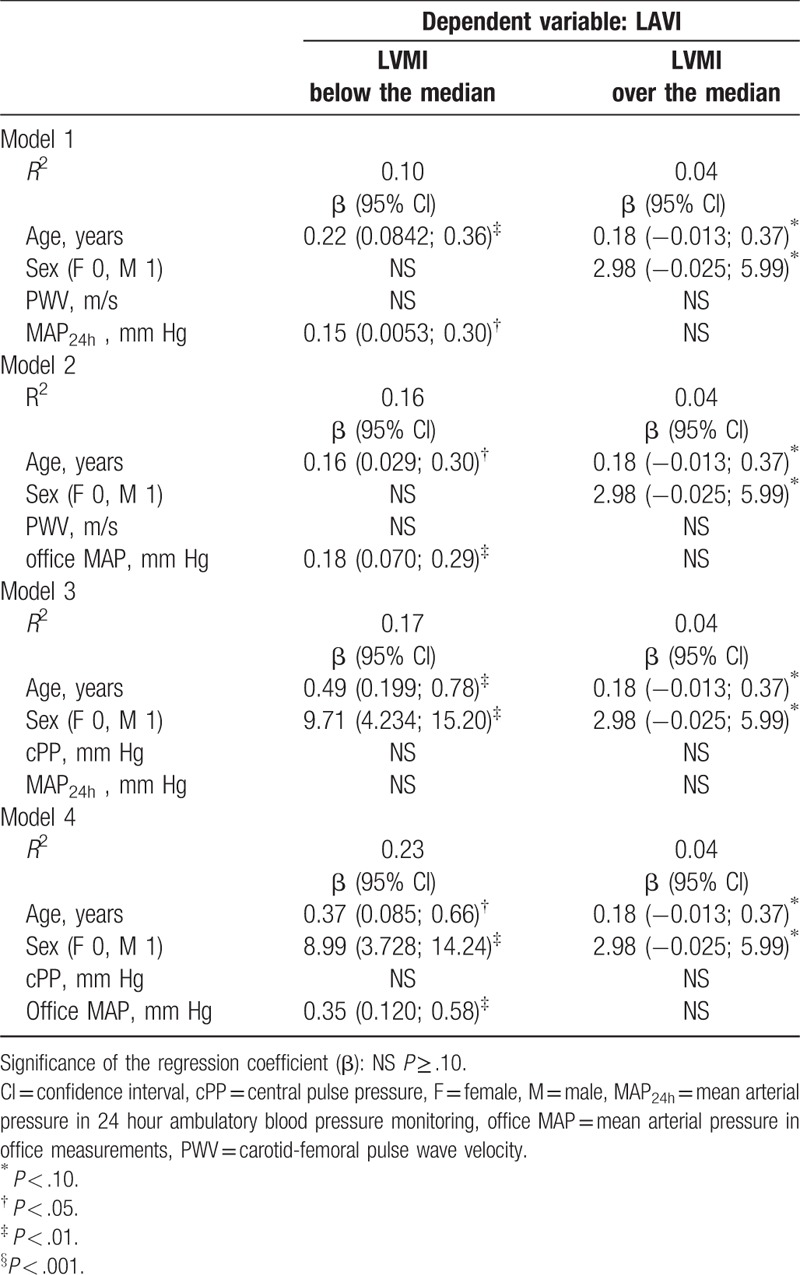
Correlates of left atrial volume index (LAVI) in subgroups with left ventricle mass index (LVMI) above and below sex-specific median.

## 4 Discussion

In this cross-sectional study, we found that the LV mass and function are the main determinants of LAVI. However, in persons with lower LV mass, LAVI depends on mean arterial pressure, but not the pulse pressure.

Considering arterial hypertension prevalence in the general population (reaching an average of 30%)^[33]^ and the prevalence of left atrium enlargement among hypertensive subjects (over 20%),^[34]^ arterial hypertension is probably the most common cause of LAE.

Elevated blood pressure induces left atrial enlargement directly or via LV hypertrophy and, consequently, diastolic dysfunction. Thus, for better discrimination of these 2 mechanisms, we divided our study group into 2 subgroups below and over the LVMI median value. The group with LVMI above median value was characterized by older age, higher BMI, higher prevalence of arterial hypertension, and diabetes. All of the above-mentioned variables or clinical conditions promote the development of LV hypertrophy.^[35]^

Moreover, the group with higher LVMI had higher blood pressure parameters (office, ambulatory) and indices of arterial stiffness-carotid-femoral PWV and central aortic blood pressure, comparing to the group presenting lower LVMI. Echocardiographic parameters of LV diastolic function were expectedly worse in the group with LVMI over the median value. Each cardiovascular variable listed above, including LVMI, may contribute to LA enlargement.^[22,23,36,37]^ The consequence of differences listed above is the higher LAVI in the group close to diagnosis of LV hypertrophy than in the group with LVMI below median value.

In both groups, LAVI was in significant positive correlation to LV mass and LVMI. Similar results were obtained for the association between LA enlargement and the LV hypertrophy by Katayama and coworkers^[38]^ in a group of adults with normal LV systolic function. Katayama et al^[38]^ suggested that elevated LV mass influences more directly left atrium through the elevation of LV filling pressure than the presence of hypertension. This conclusion is consistent with our results in the group with increased LVMI, where neither any blood pressure parameters, nor arterial stiffness correlated with LAVI. In contrary, in our group with lower LVMI blood pressure and arterial stiffness parameters remained in significant relation with LAVI. The findings might imply that at certain stage of LV hypertrophy progression, BP values are of less importance for further LAE and its pathological consequences like arrhythmia and systemic embolism, than LV hypertrophy itself. In the interim, BP parameters may influence LAVI, therefore BP control, independently of antihypertensive drug used, should prevent LA enlargement. Once LV hypertrophy develops along with its hemodynamic consequences for the LA, more attention should be paid to the antihypertensive medication choice. Priority should be given to those with evidence of cardiac hypertrophy prevention in order to counteract late LAE complications.^[27]^

Similarly to our results Lantelme et al^[21]^ and Xu et al^[39]^ found significant relation between arterial stiffness (i.e. carotid-femoral PWV) and LA diameter, while Janwanishstaporn and Boonyasirinant^[40]^ reported the correlation for LAVI, all in the groups of hypertensive subjects.

Many studies confirmed the link between different blood pressure parameters and LA diameter or volume.^[9,23,26,36]^ Some of them indicated that after inclusion of LV mass in the multivariable regression models, the relations between blood pressure variables and LA size were no longer statistically significant.^[23]^

In other studies, like the Losartan Intervention For Endpoint Reduction in Hypertension (LIFE) study, systolic blood pressure was an independent covariate of LA enlargement in patients with LV hypertrophy, but the odds ratio for this relation was only 1.01 (95%CI: 1.002–1.02). For comparison, the odds ratio in the relation between LV hypertrophy and LA enlargement in LIFE study was 2.46 (95%CI: 1.76–3.45).^[9]^

According to our results from a selected sample of the general population, we suggested that in subjects who are older, with a higher prevalence of arterial hypertension, and with increased LVMI, LV mass is crucial for LA enlargement. In younger subjects with a lower blood pressure, lower prevalence of hypertension and other comorbidities, like diabetes and consequently, lower LVMI, blood pressure and arterial stiffness parameters are more significant for LA enlargement. The last finding is of special importance when we consider LA enlargement as an early sign of hypertensive heart disease. Su et al., in a group of patients with early stages of arterial hypertension, found a 57% incidence of LA enlargement.^[37]^

In the group of patients where LVMI is not the predominant variable determining left atrium volume, it is a point of interest, which component of blood pressure is closer related with left atrium volume index.

Vaziri et al^[23]^ proved a significant correlation between LA size and BP parameters including SBP, PP, and MAP. However, after adjustment for BMI and age, only SBP and PP did have a significant contribution to LA size.

Among hypertensive patients in the study of Cuspidi and coworkers in multivariate correlation analysis systolic and pulse pressure correlated positively and diastolic blood pressure negatively with left atrium size. MAP value was not included in this analysis.^[26]^

Similar results indicating PP as a determinant of LA enlargement were demonstrated by other investigators.^[24,25]^ In diabetic subjects, Zapolski and Wysokinski^[36]^ demonstrated a correlation between PP and LAVI and lack of correlation between LAVI and aortic DBP, which is mainly reflected the steady component of blood pressure.

Contrary to the results cited above, in our group of patients with LVMI below median value, LAVI was in significant association with MAP values, but not with PP values. Regression models we fitted supported the clear distintion of relevant BP components influence on LAVI. We namely assumed introduction only 1 BP variable measured in given settings and representing steady or pulsatile BP component to eliminate multicollinearity (e.g., MAP_office_ measured using Omron device and cPP measured by SphygmoCor). Different approach to regression models’ construction (including more than 1 BP variable in category) was proposed by Mulè and coworkers^[41]^ who presented interesting observation of BP components predicting LV hypertrophy.

MAP measurement methodology may substantially influence the study results, thus, we employed more than 1 BP evaluation method. MAP_24h_ is considered to be more informative as it is derived from multiple, circadian, core measurements, unlike MAP_office_. Importantly, MAP parameters measured in different settings were proved to be significant predictors of LAVI, however MAP_office_ was considerably stronger. The models including MAP_office_ explained 16% to 23% of LAVI variability, comparing to 10% for MAP_24h_.

MAP, considered as a steady component of BP from the physiological point of view, is the function of cardiac output, systemic vascular resistance, and central venous pressure. In subjects with normal LV systolic function and cardiac output, as in the case of our study, the main factor determining MAP is systemic vascular resistance. As evident from the formula for MAP, MAP = DBP + 1/3 (SBP-DBP). Both, SBP and DBP, are in strong positive association with cardiac afterload, one of the most important factors responsible for LV hypertrophy development. Thus, it is not surprising that MAP was associated positively with LV wall thickness and negatively with LV diastolic function in a large cohort in the Framingham Heart Study. In Framingham study PP was associated, similarly to MAP, with LV diameter and wall thickness, but additionally with LV systolic function.^[42]^ Darne and coworkers reported stronger relation of LV hypertrophy with steady than pulsatile BP component.^[43]^ In essential hypertensives, older than 50 years, Mulè and coworkers^[41]^ found significant independent association between LV hypertrophy and both PP_24h_ and MAP_24h_, however in younger than 50 years only MAP was associated with LVH.

Because LAE is usually the consequence of LV hypertrophy and impaired LV filing we may expect similar stronger relation between MAP and LAVI than PP and LAVI.

Of note, in the Physicians’ Health Study MAP next to SBP and DBP strongly predicted cardiovascular diseases (CVD) in younger man, whereas either average SBP or PP predicted CVD among older.^[44]^ Calculation of LAVI in early stages of arterial hypertension, as well as in patients with low estimated cardiovascular risk, with no signs of LV hypertrophy or arterial stiffness (low PWV and PP values), may provide new information about their cardiovascular risk.

Our results should be considered within the context of the limitations of our study. The study group consisted of participants from a specified age range (40–65 years) which was optimal to evaluate the early signs of heart remodeling. For this reason, the results of the study should be extrapolated for the general population with caution. We used median value of LVMI to distinguish groups with or without a tendency for LV hypertrophy, which is more appropriate for the general population than using defined cut-off points recommended by guidelines for hypertensive patients.^[27]^ Of note, 50% of study group was diagnosed for arterial hypertension. Expectedly, hypertension was more prevalent in the group with higher LVMI, however the frequency and type of antihypertensive medication used was comparable; thus we did not expect any differences in the results obtained secondary to the medication used, which was confirmed in sensitivity analysis.

## 5 Conclusions

LV mass and function are the main determinants of LAVI. However, in persons with lower LV mass, LAVI depends on the steady component of blood pressure, but not the pulsatile one. Increased LAVI reflects early changes in response to systemic blood pressure elevation.

## Acknowledgments

We are sincerely grateful to Mrs Elzbieta Zietara and her team for the organizational support and technical assistance with the echocardiographic examination, arterial stiffness, and ambulatory blood pressure measurements. Mr Daniel Rabczenko provided us statistical expertise, for which we thank.
